# Peripheral Blood Monocyte Sirt1 Expression Is Reduced in Patients with Coronary Artery Disease

**DOI:** 10.1371/journal.pone.0053106

**Published:** 2013-01-29

**Authors:** Alexander Breitenstein, Christophe A. Wyss, Remo D. Spescha, Fabian C. Franzeck, Danielle Hof, Meliana Riwanto, Matthias Hasun, Alexander Akhmedov, Arnold von Eckardstein, Willibald Maier, Ulf Landmesser, Thomas F. Lüscher, Giovanni G. Camici

**Affiliations:** 1 Cardiology, Cardiovascular Center, University Hospital Zurich, Zurich, Switzerland; 2 Cardiovascular Research, Physiology Institute, University of Zurich, Zurich Switzerland; 3 Center for Integrative Human Physiology (ZHIP), University of Zurich, Zurich, Switzerland; 4 Institute of Clinical Chemistry, University Hospital of Zurich, Zurich, Switzerland; University of Leicester, United Kingdom

## Abstract

**Background:**

Inflammation plays a key role in atherosclerosis. Sirt1 regulates transcription factors involved in inflammatory processes and blunts atherosclerosis in mice. However, its role in humans remains to be defined. This study was therefore designed to investigate the role of Sirt1 in the development of atherosclerosis.

**Methods and Results:**

48 male subjects admitted for cardiac catheterization were subdivided into healthy subjects, patients with stable coronary artery disease (CAD), and with acute coronary syndromes (ACS). Monocytes were isolated and Sirt1 mRNA levels were determined. Sirt1 gene expression was higher in healthy subjects as compared to patients with CAD or ACS (*P*<0.05), respectively. Interestingly, HDL levels correlated positively with Sirt1 expression. Thus, HDL from the three groups was isolated and incubated with THP-1 monocytes to determine the effects of HDL on Sirt1 protein in controlled experimental conditions. HDL from healthy subjects stimulated Sirt1 expression in THP-1 monocytes to a higher degree than HDL from CAD and ACS patients (*P*<0.05). Paraoxonase-1 (PON-1), a HDL-associated enzyme, showed a reduced activity in HDL isolated from CAD and ACS patients as compared to the controls (*P*<0.001).

**Conclusions:**

Monocytic Sirt1 expression is reduced in patients with stable CAD and ACS. Experiments on THP-1 monocytes suggest that this effect is HDL-dependent and is mediated by a reduced activity of HDL-associated enzyme PON1.

## Introduction

Coronary artery disease (CAD) represents a major health burden and accounts for the majority of deaths in the Western civilisation. CAD is associated with atherosclerotic plaques, while endothelial erosion and plaque rupture with a superimposed thrombus is the underlying pathophysiological process leading to acute coronary syndromes (ACS) [1]. There is growing evidence that both development of atherosclerotic lesions as well as atherothrombotic complications are driven by inflammatory processes initiated by modified lipoproteins and other risk factors [2]. Circulating monocytes are thereby critically involved since monocyte infiltration into the arterial vessel wall represents the initial step of atherosclerotic plaque formation [3].

The mammalian silent information regulator-two 1 (Sirt1) is a NAD^+^-dependent class III histone deacetylase [4]. In addition to maintaining chromatin structure, Sirt1 regulates the activity of various transcription factors which act as key players in inflammatory processes [5]. In line with this, there is growing evidence that Sirt1 is critically involved in CAD. Indeed, in a mouse model of atherosclerosis, partial deletion of Sirt1 in bone marrow-derived macrophages promotes the development of atherosclerotic plaques [6].

Based on the above, the question arises whether Sirt1 expression in monocytes of patients suffering from CAD or ACS is altered as compared to subjects without coronary artery disease.

## Methods

### Patient Data

Male subjects aged 48–65 years admitted to the cardiac catheterization unit at the University Hospital Zurich between December 2009 and September 2011 for coronary angiography were included in the study. Patients suffering from diabetes mellitus, as well as individuals diagnosed with an active neoplastic, infectious of autoimmune disease were not included in the study population. For the CAD group, a history of ACS <6 months prior to the study was considered as an exclusion criterion.

### Ethics Statement

Ethical approval was granted by the institutional ethical committee (Kantonale Ethikkommission Zürich). All subjects signed an informed consent form.

### Data Acquisition Procedures

Coronary atherosclerosis was determined by quantitative coronary angiography. Criteria for study group assignment were (a) no angiographically identifiable coronary stenosis (>20%) or diffuse coronary atherosclerosis for the control group, (b) at least one stenosis ≥75% in either left, circumflex or right coronary artery for the CAD group and (c) either a ST-elevation (typical chest pain and two ST segment elevations ≥0.1 mV) or a non-ST-elevation (typical chest pain with four-fold elevation of Troponin T) myocardial infarction, both being admitted for percutaneous coronary intervention, for the ACS group. Previous medication was not discontinued for this study. Blood pressure was measured in horizontal position using automatic blood pressure meters, and body weight as well as height were measured on hospital admission. Smoking was defined as cigarette consumption of >10 cigarettes/day. Blood parameters were measured on automated routine analyzers.

### Isolation of Peripheral Blood Monocytes

Blood was collected in Ficoll tubes (Vacutainer CPT, BD Diagnostics) and centrifuged for 20 min at 1800 g and room temperature. The turbid white layer above the Ficoll containing the mononuclear blood cells was transferred to a clean tube and washed twice with PBS. Subsequently, monocytes were isolated using magnetic CD14-coated beads and magnetic activated cell sorting (MACS). Purity of isolated monocytes was tested by flow cytometry in cells stained with a fluorescin-labeled CD14-antibody. Finally, isolated cells were resuspended in TRIzol reagent (Invitrogen) and the total RNA extract was stored at −80°C.

### Measurement of Sirt1 mRNA Levels by Real-time PCR

Total RNA was extracted from PBM with 1 mL TRIzol Reagent (Invitrogen) as described [7]. Conversion of total cellular RNA to cDNA was carried out with Moloney murine leukemia virus reverse transcriptase and random hexamer primers (Amersham) in a final volume of 33 µL using 4 µg of RNA. The total cDNA pool obtained served as template for subsequent PCR amplification with primers specific for human Sirt1 (Forward primer sequence 5′-TGAGGCACTTCATGGGGTATGG-3′, reverse primer sequence 5′-TCCTAGGtTGCCCAGCTGATGAA-3′). Real-time PCR amplification was performed in an MX3000P PCR cycler (Stratagene) using the SYBR Green JumpStart kit (Sigma) in 25 µL final reaction volume containing 2 µL cDNA, 10 pmol of each primer, 0.25 µL of internal reference dye, and 12.5 µL of JumpStart Taq ReadyMix (buffer, dNTP, stabilizers, SYBR Green, Taq polymerase, and JumpStart Taq antibody). The amplification program consisted of 1 cycle at 95°C for 10 minutes, followed by 40 cycles at 95°C for 30 seconds, 57°C for 1 minute and 72°C for 1 minute. A melting curve analysis was performed after amplification to verify the accuracy of the amplicon. Ribosomal S12 RNA served as loading control.

### Measurement of Serum Interleukin-6 (IL-6)

IL-6 protein expression was determined using chemiluminescence immunoassays from DPC (Bühlmann), on an Immulite 2000 analyzer with a maximal variation coefficient of 11%. 50 µl of plasma from each patient was used.

### HDL Isolation

HDL from the three patient groups was isolated by sequential ultracentrifugation (*d* = 1.063–1.21 g/mL) using solid potassium bromide (Merck) for density adjustment as described previously [8], [9]. Blood samples were processed within 1 hour after collection.

### Cell Culture Experiments

THP-1 cells (LGC Promochem) were cultured as described previously [10]. Briefly, cells were grown to confluence (2×10^6^ cells per mL) in 3 cm cell culture dishes with RPMI 1640 containing 10% fetal calf serum (FCS) and rendered quiescent for 24 hours in medium with reduced amount of FCS (0.5%) before pretreatment with isolated HDL (10 µg/mL). HDL was added 6 hours before cell lysis.

### Western Blotting

Cells were lysed in a buffer solution (50 mM Tris pH 7.5, 150 mM NaCl, 1 mM EDTA, 1 mM NaF, 0.1 mM Na_3_VO_4_, 0.5% NP-40, 10 µg/µL Aprotinin, 10 µg/µL Leupeptin, 1 mM PMSF, and 1 mM DTT). 25 µg protein were loaded per lane, and 10% SDS-PAGE was performed. Proteins were transferred to PVDF membranes (Millipore) by semidry transfer. An antibody against human Sirt1 (Santa Cruz) was used at 1∶2′000 dilution. An antibody against glycerinaldehyd-3-phosphat-dehydrogenase (GAPDH, 1∶20′000, Sigma) was applied as loading control.

### Measurement of PON1 Activity

Arylesterase activity of HDL-associated paraoxonase was measured by spectrophotometry using the substrate phenylacetate.

### Statistical Analysis

Continuous variables are expressed as median (interquartile range) and frequencies for categorical variables. The inclusion of 14 patients in each group, for a *P*-value of 0.05 (two-tailed; assuming a difference in densitometric unit of 0.1 and a standard deviation of 0.08), has the power to detect a significant difference of 91%. Clinical data was analyzed by the χ^2^ test for categorical and by the Mann-Whitney-U test for continuous data. Spearman’s correlation analysis was used to assess correlation between variables. For Western Blotting, ELISA, and real-time PCR nonparametric one-way ANOVA with Bonferroni post-hoc analysis was performed. The level of significance was defined as a two-tailed *P* value <0.05. All statistical analyses were performed with SPSS Statistics 19.0 for Windows (SPSS, Inc. 2010).

## Results

### Patient’s Characteristics

**Figure 1:**
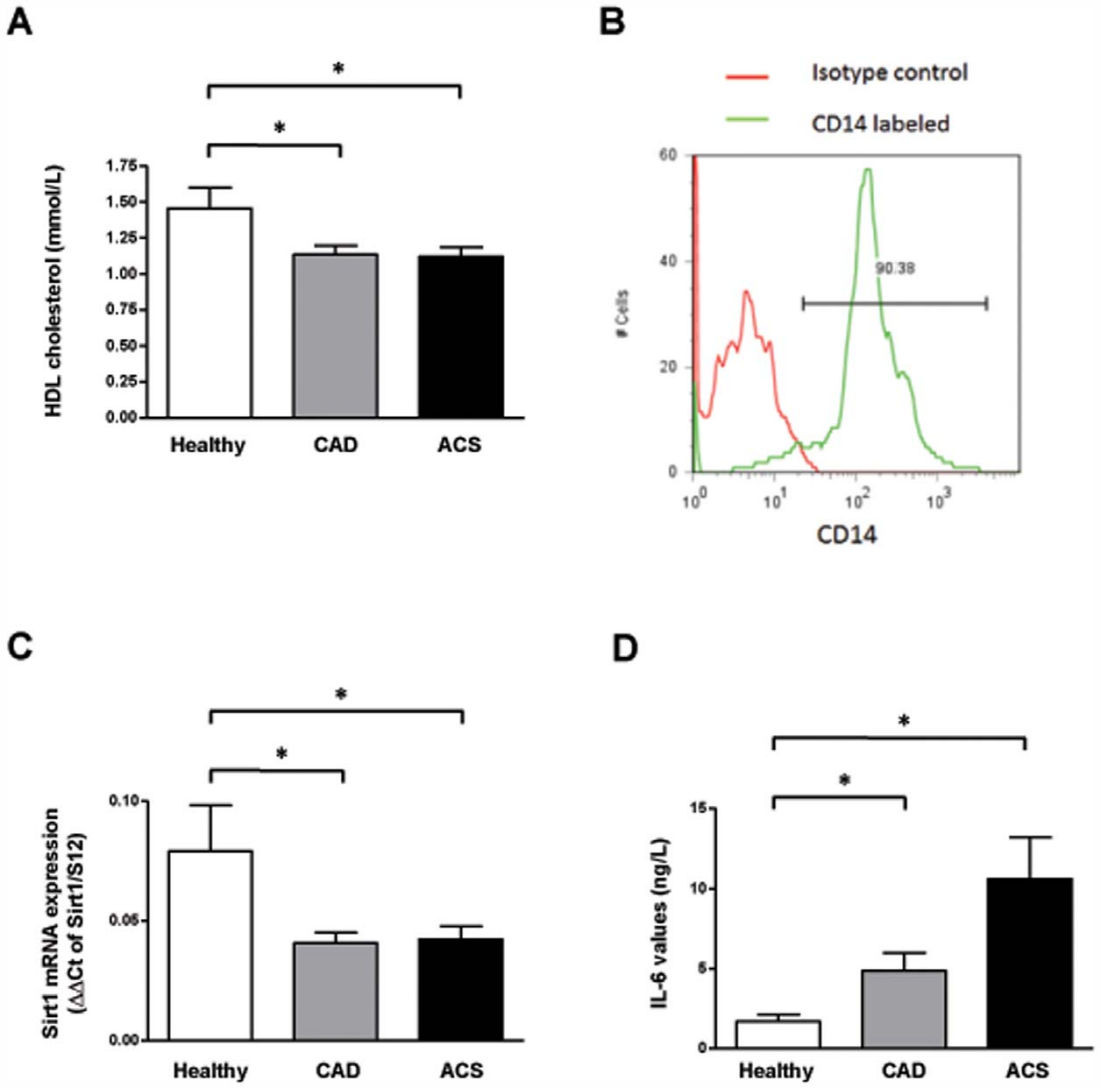
Sirt1 expression is reduced in patients suffering from atherosclerotic disease. A. HDL levels are reduced in patients suffering from CAD or ACS as compared to the healthy controls. **B.** Flow cytometry analysis demonstrates the purity of isolated monocytes. **C.** Sirt1 mRNA expression in relation to 18S rRNA in peripheral blood monocytes isolated from healthy subjects, from patients with angiographically-confirmed CAD and patients presenting with an ACS. **D.** The expression of interleukin-6 (IL-6), a negatively regulated Sirt1-dependent marker, is increased in patients with impaired Sirt1-expression.

48 subjects were included in the present study (13 age-matched, control subjects without coronary artery disease, 19 CAD and 16 ACS patients, respectively). A detailed overview of the patient’s characteristics is summarized in table 1. Briefly, medium age was 59 years and all patients were of Caucasian origin. In the CAD group, 4 subjects (21%) had one, 4 (21%) had two and 11 (58%) had three vessel disease. In the ACS group, 7 patients suffered from ST-segment elevation myocardial infarction (STEMI; 39%) and 11 of Non-ST-segment elevation myocardial infarction (NSTEMI; 61%). Myocardial infarction has been diagnosed based on findings on electrocardiography and laboratory markers of myocardial necrosis, i.e. troponin T and creatine kinase. Importantly, CRP levels were higher in patients with ACS (*P*<0.05 vs healthy and CAD). As expected, more patients in the CAD group took anti-platelet drugs and statins as compared to healthy people as well as the ACS group (*P*<0.05 for CAD vs healthy and ACS group). Interestingly, higher HDL cholesterol levels were found in the healthy group as compared to CAD and ACS patients (*P*<0.05 for healthy vs CAD and ACS group; Figure 1A).

**Table 1:** Clinical characteristics of the three patient groups.

	Control (n = 13)	CAD (n = 19)	ACS (n = 16)	*P* value
**Gender [m/f]**	13/0	19/0	16/0	NS
**Age [years]**	59±3.8	59.8±3.8	58.7±4.3	NS
**Systolic BD [mmHg]**	132±9	135±14	135±21	NS
**Diastolic BD [mmHg]**	79±10	78±7	84±16	NS
**BMI [kg/m2]**	27.1±4.2	28.0±4.2	27.6±5.1	NS
**Total cholesterol [mmol/L]**	4.6±1.2	4.3±0.7	5.3±1.1	<0.05*
**LDL cholesterol [mmol/L]**	2.6±1.2	2.5±0.6	3.5±0.9	<0.05*
**HDL cholesterol [mmol/L]**	1.4±0.4	1.1±0.2	1.1±0.2	<0.05†,‡
**CRP [mg/L]**	1.4±1.0	2.4±3.2	8.4±9.3	<0.05*
**Adipositas**	3 (23.1)	5 (26.3)	4 (25.0)	NS
**Dyslipidemia**	4 (30.8)	10 (52.6)	9 (52.9)	<0.05†,‡
**Hypertension**	5 (38.5)	11 (57.9)	11 (64.7)	<0.05†,‡
**Current smoking**	3 (23.1)	3 (15.8)	7 (41.2)	<0.05*
**Aspirin**	6 (46.2)	14 (73.7)	14 (73.7)	<0.05†,‡
**Clopidogrel**	0 (0)	7 (36.8)	7 (36.8)	<0.05†,‡
**Beta-Blocker**	4 (30.8)	9 (47.4)	9 (47.4)	NS
**ACEI/ATB**	6 (46.2)	9 (47.4)	9 (47.4)	NS
**Statins**	5 (38.5)	13 (68.4)	13 (68.4)	<0.05†,‡
**Diuretics**	2 (15.4)	3 (15.8)	3 (15.8)	NS

* 0.05* CADnts with CAD and ACS,ACS group)erosclerosis looses its cardioprotectice properties.**

* ACS vs CAD.

† Healthy vs CAD.

‡ Healthy vs ACS.

### Sirt1 Expression is Decreased in Patients with Coronary Artery Disease

Flow cytometry analysis of the isolated monocytes stained for CD14 demonstrated the high degree of purity of the isolated cells (Figure 1B). Sirt1 mRNA levels in monocytes were significantly higher in healthy controls (0.0791±0.019; n = 13; Figure 1C) as compared to CAD (0.041±0.004; n = 19; *P*<0.05 vs control) and ACS patients (0.042±0.005; n = 18; *P*<0.05 vs control). However, expression levels of Sirt1 did not differ significantly between CAD and ACS patients (*P* = NS).

To confirm Sirt1 pathway inhibition in CAD and ACS patients, expression of Sirt1-negatively regulated downstream target interleukin-6 (IL-6) [11] was determined. In line with our expectation, IL-6 protein expression was upregulated in the same patient groups (n = 10–12; *P*<0.05; Figure 1D).

### HDL from Healthy Patients Increases Monocyte Sirt1 Expression, while HDL from CAD and ACS Patients does not

**Figure 2:**
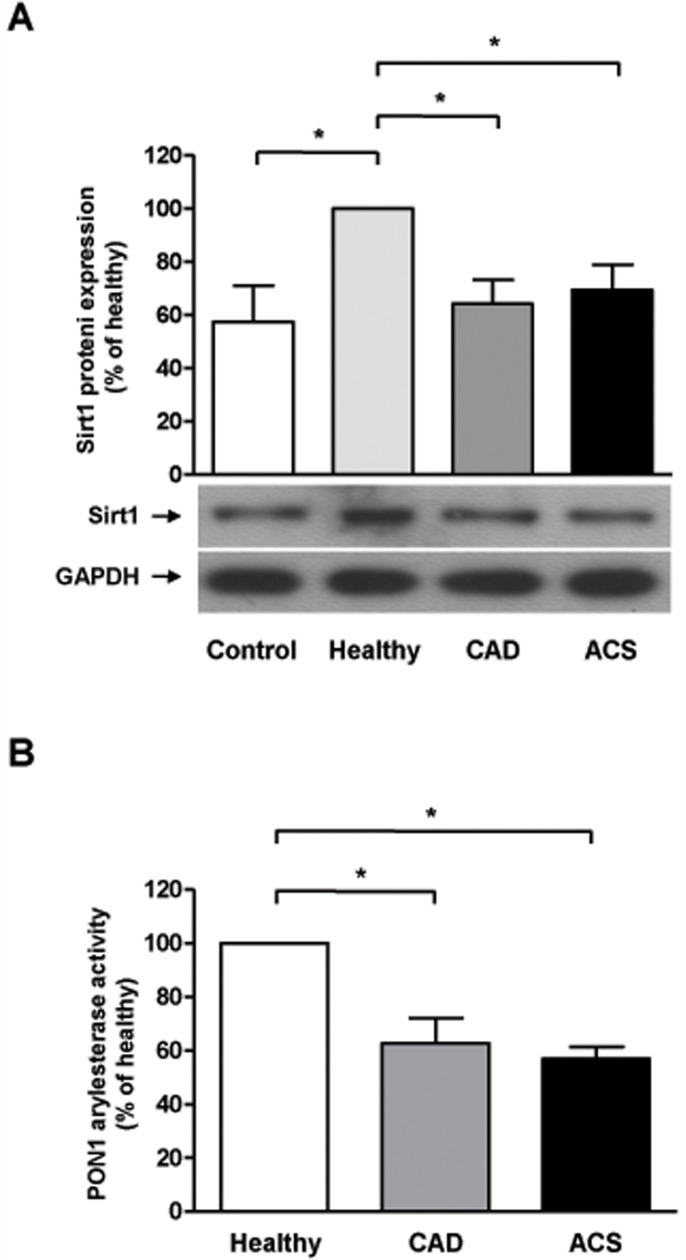
HDL regulates Sirt1 expression. A. Sirt1 protein expression in THP1 cells after incubation with HDL from healthy subjects or patients suffering from CAD and ACS, respectively. **B.** PON1 function, as measured by PON1 arylesterase activity, is significantly reduced in the CAD and ACS group.

In order to assess potential direct effects of HDL on Sirt1, THP-1 cells, a monocyte cell line, were incubated with HDL isolated from healthy subjects, CAD or ACS patients, respectively, and Sirt1 protein expression analyzed by Western blotting. As shown in figure 2A, HDL from healthy subjects increased Sirt1 protein expression as compared to vehicle-treated cells (n = 5–11, *P*<0.05 vs control; Figure 2A), while HDL from to CAD and ACS did not (n = 9–12; *P* = NS vs control, Figure 2A).

### Paraoxonase 1 (PON1) Activity is Reduced in CAD and ACS Patients

PON1 is a HDL-associated enzyme which has been demonstrated to affect atherosclerosis [12]. Therefore, PON1 activity was measured in HDL from healthy subjects, CAD and ACS patients. As shown in Figure 2B, PON1 activity is significantly reduced in the CAD and ACS group as compared to the healthy control (n = 9; *P*<0.001; Figure 2B).

## Discussion

Here we demonstrate for the first time that the expression levels of the longevity gene Sirt1 in peripheral blood monocytes is reduced in patients with stable CAD and in those with ACS, respectively, as compared to subjects without angiographically demonstrable CAD. Interestingly, Sirt1 levels correlated positively to HDL levels in all three study groups. In line with this observation, THP-1 monocytic cells incubated with HDL isolated from healthy subjects displayed increased Sirt1 protein expression as compared to THP-1 monocytic cells incubated with HDL from the CAD or ACS group. Furthermore, PON1 activity was reduced in HDL from CAD and ACS groups as compared to healthy subjects, indicating that PON1 activity is required in order for HDL to stimulate Sirt1 expression.

Sirt1, a key regulator of inflammatory processes, reduces atherosclerosis in different animal models [6], [13]. Importantly, Sirt1 in endothelial cells and Sirt1 from bone marrow-derived macrophages are both involved in this anti-atherogenic effect. However, whether Sirt1 also displays anti-atherogenic properties in humans and in CAD patients in particular, has not yet been shown. In the present study, we report that monocyte Sirt1 gene expression is decreased in patients suffering from coronary atherosclerosis; in line with this, expression of Sirt1-negatively regulated downstream target interleukin-6 (IL-6) [11] was increased in the same patient groups. Although, as shown previously, CRP was elevated in ACS patients compared to stable CAD, acute activation of the immune system – at least as reflected by an elevated C-reactive protein in the ACS group – does not seem to play a role in the regulation of Sirt1 expression. This finding is in line with results from *in vitro* studies, were acute stimulation of cultured endothelial cells with inflammatory cytokines did not change Sirt1 protein expression [5]. However, whether the impaired Sirt1 levels in patients suffering from coronary atherosclerosis are a result of the atherosclerotic process itself or vice versa, cannot be answered by this study.

Interestingly, Sirt1 expression correlated positively with the plasma levels of HDL cholesterol suggesting a regulatory role of HDL on Sirt1 expression. Indeed, epidemiologically HDL plasma levels are inversely related to myocardial infarction and stroke even in patients treated with a statin [14]. In contrast to the well-known pro-atherogenic LDL cholesterol [15], [16], HDL can exert profound vascular protective functions [17]. Of note, recently published data from our institution examining the endothelial protective role of HDL showed that its beneficial properties are lost in specific subset of patients indicating that HDL plasma levels may not reflect the functional properties of the lipoprotein [9], [18]. Indeed, HDL from CAD and ACS patients, unlike that of healthy subjects, fails to stimulate endothelial nitric oxide production in human aortic endothelial cells, underscoring a loss of its anti-atherogenic properties [9]. Our *in vitro* data are in line with these recent findings, since HDL from healthy subjects increased Sirt1 protein expression in THP-1 cells, while matching concentrations of HDL from CAD and ACS patients failed to do so. Thus, in this setting, not only the reduced plasma levels of HDL in patients with CAD and ACS must have contributed to the reduced Sirt1 expression, but – since these data have been obtained with matching HDL concentrations in all three experimental groups – also a biological dysfunction of the HDL particles associated with CAD and ACS.

PON1 is a HDL-associated antioxidant enzyme that has been shown to reduce the development of atherosclerotic lesions in mice [19], while low activity of PON1 in humans is associated with the risk of CAD and cardiovascular events [12]. Interestingly, PON1 activity was reduced in HDL isolated from CAD and ACS patients in our study. Loss of PON1 activity results in a reduced level of endothelial nitric oxide synthase (eNOS) activity [9], which is an upstream target of Sirt1. Hence, normal PON1 activity appears essential for Sirt1 expression.

In summary, we here demonstrate for the first time that in patients with CAD and ACS monocyte Sirt1 expression levels are markedly decreased as compared to healthy individuals. While treatment of a human monocyte cell line with HDL isolated from healthy people raised Sirt1 expression, HDL obtained from patients with CAD and ACS completely failed to do so, probably due to the loss of PON1 activity. The reduced Sirt1 levels in CAD and ACS therefore appear to be related both to reduced HDL levels and HDL dysfunction.
